# Fertility increases women’s desire for conspicuous luxury

**DOI:** 10.1371/journal.pone.0354126

**Published:** 2026-09-02

**Authors:** Aekyoung Kim, Kristina M. Durante, Juliano Laran, Lambrianos Nikiforidis, Vladas Griskevicius

**Affiliations:** 1 Jeonbuk National University, Jeonju-si, Jeollabuk-do, Republic of Korea; 2 Rutgers Business School, Newark, New Jersey, United States of America; 3 Schema Behavioral Science, Newberry, Florida, United States of America; 4 School of Economics and Business, SUNY Oneonta, Oneonta, New York, United States of America; 5 Carlson School of Management, University of Minnesota, Minneapolis, Minnesota, United States of America; Indira Gandhi National Tribal University, INDIA

## Abstract

This paper examines hormonal fluctuations associated with fertility as one of the reasons behind women’s desire for luxury. We suggest that women engage in conspicuous luxury consumption during ovulation, when they have a physiologically activated mating goal, because doing so helps women be noticed by men. Being noticed by as many men as possible increases the likelihood of attracting a desired partner for a romantic relationship. The finding that ovulation—the time of peak fertility—increases women’s desire to purchase conspicuous luxury goods challenges previous research suggesting that women use luxury to signal high standards and limit their pool of potential partners. We reconcile this apparent contradiction by proposing a two-phase mating process: women first seek to attract a broad pool of potential partners (attraction phase), then select from among them for a romantic relationship (selection phase). These findings have benefits for researchers’ understanding of how ovulation changes women’s behavior towards men, and how mating motives can be a driver of the desire for luxury and conspicuous consumption in general.

Consumers spend billions of dollars each year on publicly visible high-end goods such as designer clothes and accessories, jewelry, and luxury cars [1]. Past research has examined this behavior from a status perspective, as consumers often communicate their status through luxury goods that signal wealth and prestige [2–4]. Importantly, women account for nearly half of the spending on luxury goods and services [5]. Of course, some of this spending is due to seeking status, but it raises questions about other reasons why women may engage in conspicuous luxury consumption. We contend that one factor influencing women’s luxury spending is the change in a woman’s fertility across the ovulatory cycle, which influences their desire to be noticed.

This research aims to show that conspicuous luxury consumption is a means women use to attract mates near ovulation (i.e., high fertility), when estrogen levels increase and induce a nonconscious mating goal [6–7]. When a woman seeks to attract a potential mate, luxury consumption is a way for her to be noticed by men. Prior work finds that women near ovulation use different strategies to attract men [8–9]. One strategy is to dress in a sexy way [10], which is part of a class of behaviors aimed at increasing the chance of being noticed. Here, we propose an alternative strategy that ovulating women may engage in to be noticed by men, which involves conspicuous luxury consumption. We predict that women near ovulation are more likely to prefer luxury products (e.g., a designer bag or shoe) and display luxury in a conspicuous way, such as by wearing clothes with large logos that others will notice. To investigate this prediction, we conduct studies using urine samples to measure hormone levels and a survey method that estimates a woman’s current phase in the menstrual cycle.

This investigation offers new insights relative to previous research. First, previous research posits that women use luxury to signal high standards and deter a large number of possibly undesirable partners [11]. We show that women may use luxury to be noticed by a large number of possible partners, which challenges the idea of using luxury to deter partners. Second, much of previous research explores the status-seeking behavior of men, which can help them attract women [12]. While status-seeking is not their main motive, we show that women may also use signals typically associated with status-seeking (luxury goods) in order to reach their goal of being noticed. Finally, this research shows a novel factor – high fertility – that has not been previously explored as an influence on luxury consumption.

## Fertility, being noticed, and attracting mates

Women are most fertile near ovulation. When women are near the ovulatory phase of the menstrual cycle (i.e., day 14 of a 28-day cycle), they experience increased sexual desire [13], and may engage in behavior aimed at outshining other women to attract mates. For example, women near ovulation feel more attractive and socialize more [14–16]. Ovulating women also make suboptimal choices focused on doing better than other women [17] and seek sexy outfits when attractive women are around as competitors [8–10]. Thus, when women are near ovulation, and therefore fertility is high, they change the way they behave towards men and other women.

Recent research indicates that women adopt strategies to decrease the number of partners who pursue them, trying to attract few, desirable partners. In order to achieve this “mate screening” goal, women use luxury (e.g., prefer high-end brands) to signal their high standards and deter undesirable partners [11]. This is understandable, as this behavior stems from the evolved motivation to attract the highest-quality mate possible. However, these findings stand in contrast to findings showing that women seek to attract a sizeable number of men that they can evaluate for a possible relationship. For example, ovulating women modify their behavior before having sex or engaging in a relationship so they can expand the pool of potential partners they have to choose from [18].

We propose that this discrepancy results from nuances in how relationships develop, and the failure of previous research to distinguish between two phases. The first phase of the process of selecting men for sex or a long-term relationship is to generally attract men. Then, women can make a choice about which mate is most suitable. The initial attraction phase is akin to casting a wide net, whereas the selectivity phase is akin to surveying the net results. Indeed, the research looking at mate screening investigated contexts where the second phase occurs, such as a dating website or a party where women were “receiving an overwhelming amount of attention from men” (i.e., women were trying to be selective) [11].

This distinction between (a) an attraction phase and (b) a selection phase is important. It means that, before women become selective, they attract the attention of as many men as possible, so they can later be selective (i.e., make a choice). The idea of an attraction phase is consistent with findings that a mating goal leads women to dress sexier to attract mates [8,10,19], and a recent meta-analysis shows that this effect has strong evidential value [20]. Thus, women may engage in behavior to ensure there are enough men attracted to them so they can select from a pool of candidates that is sizeable enough.

## Being noticed and conspicuous luxury

We contend that women who are near ovulation try to attract as many men as possible so they can select the best available mate (i.e., cast a wide net) [21]. A key part of attracting several possible mates is being noticed. It is impossible for a man to be attracted to a woman if the man does not notice the woman. Thus, the strategies women adopt in order to attract men, at a basic level, should focus on getting as many men as possible to notice them. Near ovulation, women may behave in different ways with the goal of being noticed [20]. For example, ovulation may lead women to engage in exaggerated behaviors that could draw attention to them, such as wearing brighter colors, making choices that are more distinctive, or telling stories and jokes that would receive attention [22–24]. Here, we propose an alternative class of behaviors that women may adopt to be noticed: conspicuous luxury consumption.

Clearly, consumers use products to communicate with and to others. This is evident in identity research showing that consumers many times use products to signal something about who they are to the people around them [25–26]. For example, they use conspicuous consumption when they want to show superiority to others in their environment [27]. The implication is that consumers assume that others will notice them for what they consume or wear and use consumption to be noticed when they believe being noticed will send a positive signal about them. An especially relevant category when it comes to signaling via consumption is conspicuous luxury consumption, which is the use of luxury goods to signal status and prestige [4,28]. This behavior makes sense as there are many positive characteristics associated with symbols of status, including wealth, respect, power, and the suggestion that a person has a high standing in society [2,25,29].

A key characteristic of luxury products is that, for them to work as signals to others, they must be highly visible. This is why consumers signal through products that are readily observable by others, such as brand-name clothing, accessories, and cars [2–3]. In fact, the more visible the signal is, the more likely that others will notice. For example, consumers may wear luxury clothes and accessories with larger logos to ensure that others will notice them [30]. The use of luxury consumption to send signals, and the fact that these signals must be as visible as possible to work, make conspicuous luxury consumption an ideal candidate behavior for someone who wants to be noticed by others.

For this reason, we propose that women near ovulation use visible signals of luxury consumption because doing so increases the chance that men will notice them, especially over other women who may not be using signals that help them be noticed. Of course, there are levels of luxury, which can vary from very expensive possessions (e.g., a yatch or mansion) to lower-ticket products (e.g., a special edition dish or product). Here, we explore the role played by luxury clothing items and accessories that are sold by brands generally considered high-end (e.g., Prada, Gucci, Polo Ralph Lauren) and that are easily recognizable by others.

## Conspicuous luxury as an effective signal to attract men

While wearing conspicuous luxury is more noticeable than nonconspicuous luxury or not wearing luxury at all [3,27,31], an important question is how adaptive this behavior is when women are near ovulation and are motivated to attract attention from men [21]. Dressing sexy, for example, is effective at attracting men. However, when it comes to luxury, it is not clear that luxury products will attract men. This question can be answered from two perspectives. From women’s perspective, the fact that more men notice them clearly increases the chance that one of them will be attracted. Unless there are other intervening variables like a woman who lacks self-confidence in her physical attributes, attracting the attention of men is good. From a men’s perspective, the issue is more complex.

Prior work has shown that materialistic men are less attracted to women’s conspicuous consumption [32]. After all, one of the associations of luxury is wealth, and men may prefer to display their own wealth to attract women [33]. Yet, many men (e.g., those who are nonmaterialistic) are agnostic toward women’s conspicuous consumption [32], and luxury has an array of positive, beneficial associations [34]. These associations can be financial, but may also involve other desirable characteristics. For example, while one of the associations of status is dominance, which is based on intimidation, another association is prestige, which is based on having desirable skills and expertise [35]. Overall, when it comes to the argument of whether men are indeed more attracted to a woman who wears visible luxury, previous research does not provide a clear answer, as the evidence for the attributions men may make about a woman who shows status is mixed.

We contend that given the fact that more men are likely to notice a woman who is wearing conspicuous symbols of luxury, the signal will be effective as long as the attention is positive or at least not negative. If luxury is not a deterrent, and conspicuous signals are noticeable, more men will notice a woman wearing visible luxury than a woman who is not wearing visible luxury. By definition, there is a higher chance of a man being attracted to a woman that he notices. In fact, it is virtually impossible for a man to be attracted to a woman that he does not notice. For this reason, we propose that using conspicuous luxury can be a reliable strategy when a woman is trying to attract men, as this will increase the number of men who are possibly attracted to her. This will allow her to later select who she prefers, be it for a casual or longer-term relationship.

**H1**: When women are near ovulation, they have higher desire for conspicuous luxury compared to when they are not near ovulation.

## Research overview and moderators

In summary, we propose that women who are near ovulation have higher desire for conspicuous luxury compared to when they are not near ovulation. Conspicuous luxury can help them be noticed by and attract men. If our conceptualization holds, it suggests three moderators. First, ovulating women will have an increased desire for visible luxury that they will consume themselves, but not luxury they recommend for other women. The implication is that ovulation should not simply make women more positive about luxury products, but rather about luxury products that they can use for their own benefit. Second, ovulating women will have an increased desire for luxury that can be seen by others, but not luxury that is consumed in private (e.g., a high-end alarm clock, a luxurious coffee maker). Third, the positive effect of fertility is mitigated when women do not believe that men notice women’s luxury consumption (e.g., designer clothes), as women should not use a signal that is not effective. Formally:

**H2**: The increase in desire for conspicuous luxury near ovulation will occur when women make decisions for themselves, but not when they make recommendations for others.

**H3**: The increase in desire for conspicuous luxury near ovulation will occur for products to be consumed in public, but not for products to be consumed in private.

**H4**: The increase in desire for conspicuous luxury near ovulation will not occur when women are led to believe that men do not notice women’s luxury consumption.

We tested our hypotheses using two different methodologies recommended as best practices for ovulatory cycle research [36]. First, we used urine applicator tests confirming the periovulatory increase in luteinizing hormone (LH), which indicates an increase in estrogen and impending ovulation. Second, we used studies that estimate fluctuations in hormonally-regulated fertility using a survey method. Study 1 demonstrates that women prefer more conspicuous luxury when they are near (vs. not near) ovulation. It also shows that this effect does not represent a general desire for conspicuous luxury, as the effect of ovulation does not occur when women make decisions for another woman. Study 2 demonstrates that ovulation increases purchase intentions for publicly visible luxury, but does not have an effect for luxury that will not be seen by other people and for nonluxury brands. Study 3 demonstrates that the effect is mitigated by a belief that men do not notice women’s luxury products.

## Study 1: The effect of fertility on choosing conspicuous luxury for oneself

Study 1 tested hypotheses 1 and 2 by using urine hormone tests to confirm ovulation [36]. To measure women’s desire for luxury, we had women draw logos of luxury brands, as the desire for conspicuous luxury products is associated with the choice of larger luxury brand logos [30]. We predicted that women would draw larger logos near ovulation compared to low fertility (hypothesis 1). In addition, we had women draw logos for items they would recommend to other women. Because bigger logos for other women do not help a woman be noticed, we predicted that logos for other women would not be larger near ovulation (hypothesis 2).

### Method

#### Participants and design.

Prior to starting the study, ethical approval had been obtained from the local institutional review board (IRB) at University of Texas at San Antonio (#13–086) to confirm the study met national and international guidelines for research on humans. Seventy female university students (*M*_*age*_ = 22.59, *SD* = 5.01) who were not on hormonal contraception participated in the study from 2013/09/23–2014/07/23. This sample size was based on best pratices for within-subject ovulatory cycle research. These best practices recommend a sample size of n = 50 using LH surge detection for 80% power to detect a medium effect size [36]. Demographic characteristics of the sample can be found in Appendix A in S1 File. They received either course credit or $30 as compensation for their time. All participants viewed written informed consent online prior to starting the survey, and consent was given by completing the survey. The study had a 2 (fertility: low vs. high fertility) by 2 (target user: self vs. another woman) within-subjects design.

#### Procedure.

To assess fertility status, we employed a method of urinalysis [8,10,37,38]. Participants came to the lab and were administered a urine applicator test (www.meditests.com, available over the counter), which detects a surge in luteinizing hormone (LH; a pituitary hormone that spikes 24–48 hours before ovulation occurs).

Each woman served as her own control because each woman completed testing twice—once at ovulation/high fertility and again at the low fertility phase of the cycle. The order depended on where she was in her menstrual cycle on the day she completed a prescreening survey over the phone. Following the screening materials presented in previous research [39], phone screening interview questions ensured that all participants were normally ovulating and not on any form of hormonal contraception or prescription medication. Specifically, only women who reported none of the following were included in the study: current or recent (last three months) use of hormonal contraceptives, irregular cycle length (i.e., cycles running less than 25 days or more than 35 days long), recent birth of a child and/or currently breastfeeding a child, experience of dramatic changes in weight, use of antidepressants, or regular cigarette smoking. Based on the phone interview, participants were scheduled to report to the lab near their expected day of ovulation or about one week thereafter.

This method provides a natural counterbalance, resulting in 37% (63%) completing low (high) fertility testing first. Participants completed the urine tests in the laboratory each day until a positive test was detected. Research assistants conducted the reading and recording of the urinalysis results in the laboratory. If a testing day fell on the weekend, the participant took the test at home, recorded the appearance of the stick after testing, and brought the test strips back to the laboratory on Monday. Research assistants confirmed the readings. Low fertility was confirmed by a post-study phone call inquiring about the exact date of participants’ next menstruation period.

At the beginning of the study, participants were told we were interested in the relationship between health and consumer preferences over the course of several days and that the urine tests measure overall health markers. They were told that they had to complete the urine test so that the research team would have a more complete picture of their overall physical health, which could influence some of the measures in the study. Note that participants were fully debriefed at the end of session 2. None of them was cognizant of the research hypotheses, and none suspected that our research was investigating the influence of ovulation.

To measure the desire for conspicuous luxury products, we used a paradigm adopted in research showing that consumers’ desire for conspicuous products is associated with a preference for larger luxury brand logos [30,40,41]. We had women draw logos on products once when they were at the ovulatory phase of their cycle—near peak fertility—and again at the luteal phase—when fertility was low (the order was counterbalanced).

Appendix B in S1 File shows the materials we used for the drawing task. As a guide, participants were given a sheet of paper with luxury brand logos (Gucci, Chanel, and Louis Vuitton). They were asked to select one of the logos to draw on two products (handbags and shoes) for themselves and for another woman (counterbalanced). When prompted to draw for themselves, they received a pencil, were asked to imagine they were bringing some products home, and were asked to draw the brand logos they would like to have on those products, following the instructions: “We are interested in your preferences for brands. Imagine that you are going shopping for the items (on the following pages) that you would like to own and take home with you today. In this task, you will be asked to draw the brand logos you would like to have on various products. By using the pencil provided, please draw on the next few pages.” When drawing for another woman, they followed the same procedure, but with the following instructions: “Another woman will complete the study after you. We would like you to recommend brands for her. Imagine that another woman will own the items you recommend, and they will take these items home with them today. In this task, you will be asked to draw brand logos you would like another woman to have on the various products. By using the pencil provided, please draw on the next few pages.”

We assessed logo size by using a caliper that observes distance in inches, and the area of each logo was calculated as height by width in square inches. The size of the logos women drew were the dependent variable. Because past research shows that preference for different-size logos is related to socioeconomic status (SES) [3], participants also indicated their agreement with the following items (1 = *Strongly Disagree*, 5 = *Strongly Agree*): (1) “I don’t need to worry too much about being able to pay my bills” and (2) “I feel relatively poor these days” (*M* = 3.11; *SD* = 1.16; 𝛼 = .70). Items were coded such that higher numbers indicated higher levels of financial status.

#### Pretest.

A pretest using 67 women confirmed that the brands we used (Gucci, Chanel, and Louis Vuitton) were perceived as luxurious. Participants were instructed to rate how luxurious (on a 9-point scale) they found three brands. The luxury brands were perceived to be more luxurious (*M* = 7.82, *SD* = 1.16; *p* < .001, Cohen’s d = 1.16, with the mean compared to 5, the midpoint of the scale). These results confirm that the brands had the desired characteristics to test our hypotheses.

### Results

**Fig 1:**
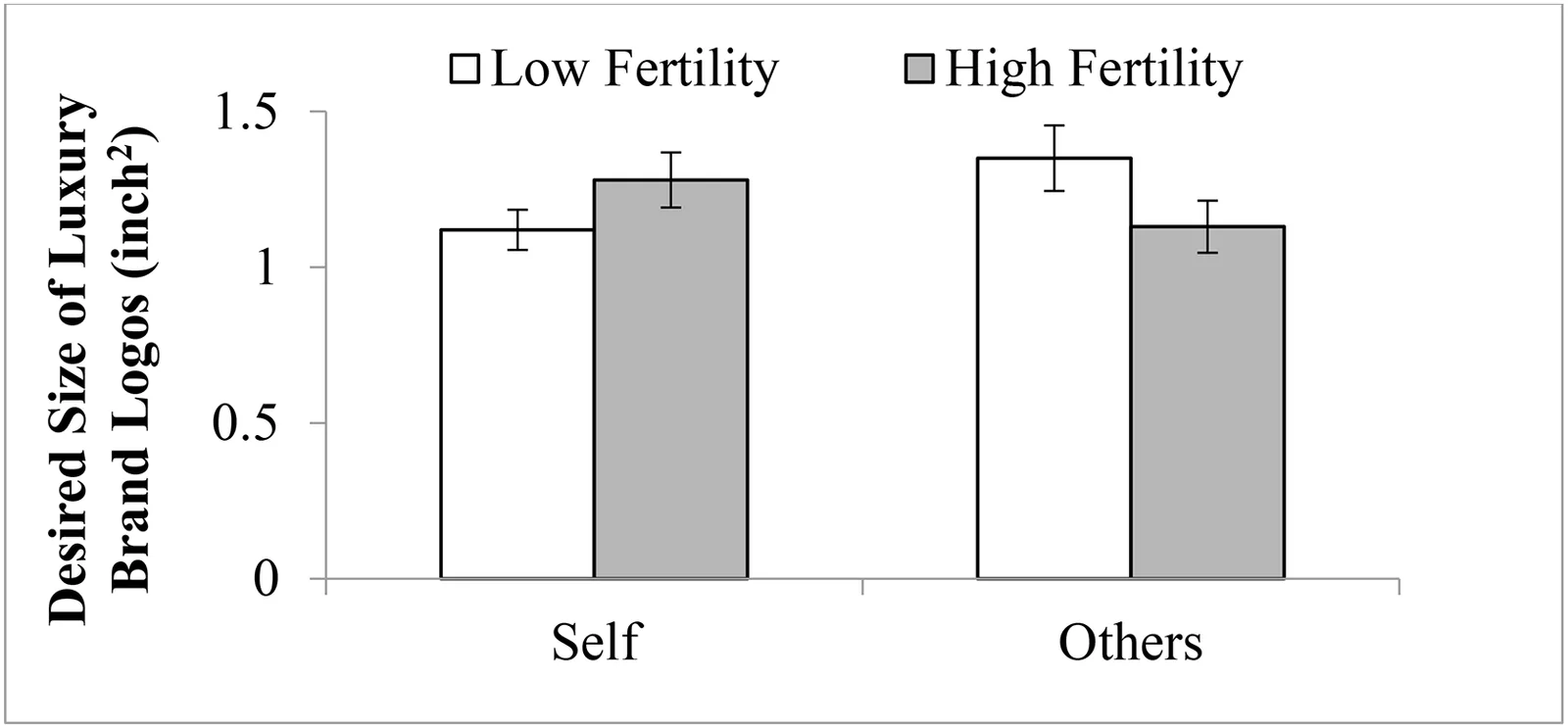
Study 1 Results.

We analyzed logo sizes using a repeated-measures ANOVA with fertility and target user as repeated factors, and SES as a covariate. Because each woman completed two study sessions (one at a low fertility and one at a high fertility point in the cycle, as confirmed via hormone tests—the most stringent method for estimating fertility), fertility is analyzed as a dichotomous variable. There was an interaction between women’s fertility level and target user (*F*(1, 68) = 16.19, *p* < .001, *η*^*2*^ = .19; the interaction held without controlling for SES (*F*(1, 69) = 5.21, *p* = .026, *η*^*2*^ = .07; see Fig 1). Consistent with hypothesis 1, when drawing logos for themselves, fertility increased logo size, as women drew significantly bigger logos in the high fertility (*M* = 1.28 inch^2^, *SD* = 1.42 inch^2^) than in the low fertility (*M* = 1.12 inch^2^, *SD* = 1.13 inch^2^) study session (*F*(1, 68) = 6.34, *p* = .014, *η*^*2*^ = .09). Further, at high fertility, women drew bigger logos for themselves (*M* = 1.28 inch^2^, *SD* = 1.42 inch^2^) compared to those for other women (*M* = 1.13 inch^2^, *SD* = .95 inch^2^; *F*(1, 68) = 4.59, *p* = .036, *η*^*2*^ = .06). Consistent with hypothesis 2, when drawing logos for other women, fertility did not increase logo size, as women drew significantly smaller logos in the high fertility (*M* = 1.13 inch^2^, *SD* = .95 inch^2^) than in the low fertility study session (*M* = 1.35 inch^2^, *SD* = 1.25 inch^2^; *F*(1, 68) = 7.88, *p* = .007, *η*^*2*^ = .10). This significant difference, which was not expected, may indicate that at high fertility women do not want other women to be noticed.

### Discussion

The findings demonstrate that women prefer more conspicuous luxury when they are ovulating, therefore supporting hypothesis 1. Supporting the idea that this occurs because conspicuous luxury helps them be noticed during ovulation, women did not draw bigger logos for other women, therefore supporting hypothesis 2. In the next two studies, we use an alternative method to measure ovulation while exploring two additional moderators. In addition, we use a larger, more diverse sample of respondents.

## Study 2: Intent to purchase conspicuous luxury

In addition to using a different method to measure ovulation, Study 2 asked women about their purchase intentions for luxury brands that would be consumed in public versus not. Consistent with hypothesis 1, we predicted that ovulation would lead to a higher desire to purchase luxury. Consistent with hypothesis 3, we predicted that this effect would occur when the luxury would be consumed in public, but not in private. This should occur because luxury that is not seen by others does not increase the chance that men will notice a woman.

### Method

#### Participants and design.

Prior to starting the study, ethical approval had been obtained from the local institutional review board (IRB) at Rutgers University (# Pro2020000942) to confirm the study met national and international guidelines for research on humans. We collected data from 1,486 women who were not currently taking hormonal contraceptives such as the pill, the patch, or hormonal IUD (*M*_*age*_ = 30.95, *SD* = 6.95) who participated for payment via Prolific from 2020/08/04–2020/08/08. This sample size was based on best practice recommendations that cross-sectional, between-subject fertility estimation use a sample size of n = 500 to detect medium size effects [36]. Demographic characteristics of the sample can be found in Appendix A in S1 File. All participants viewed written informed consent online prior to starting the survey, and consent was given by completing the survey. The study had a 2 (brand type: luxury vs. nonluxury) by 2 (consumption setting: public vs. private) between-subjects design with fertility as a continuous variable.

Although we advertised the survey for women aged 18–40, some women over the age of 40 completed the study. If we could assess fertility, they were retained in our sample just as the other women. Women were told they were participating in a study on lifestyle and consumer preferences. Based on standard practice in ovulation research [8,10,17,21,36], we applied the following screening criteria: we retained participants only if they were not taking hormonal contraceptives, had not taken hormonal contraceptives in the past three months, were not pregnant or breastfeeding, had not been sick recently (e.g., with the flu or anything more serious than a common cold), did not have an endocrine or hormonal disorder, did not suffer from a serious or chronic illness (such as cancer or diabetes), and were not currently prescribed any medication.

Accordingly, 76 women were eliminated because they were currently using an oral contraceptive or other hormonal contraceptive; another 102 because they had taken hormonal contraceptives in the past three months; another 52 for being currently pregnant or breastfeeding; another 18 for having been recently sick (e.g., with the flu); another 94 for having an endocrine or hormonal disorder; another 39 for having a serious or chronic illness (e.g., cancer or diabetes); and 167 for being on prescription medication, resulting in 938 valid responses. To increase the precision of the conception probability estimation [39], we removed participants who were unsure of their menstrual cycle dates or length (i.e., below a 6 on a 9-point scale) and had abnormal cycle lengths (i.e., < 25 or > 36 days). The final analysis included 332 women (*M*_*age =*_ 30.79, *SD* = 6.39, ranging 18–64).

#### Procedure.

Participants reported their purchase intentions of six brands by answering the question, “To what extent would you like to buy a T-shirt by [brand]?” (1 = *Not At All*, 7 = *Very Much*). Half the participants were randomly assigned to luxury brands (Chanel, Prada, Gucci, Louis Vuitton, Fendi, and Burberry), and the other half were assigned to nonluxury brands (Gap, Old Navy, JCPenney, H&M, Target, and Walmart). We randomized the order of the brands to avoid any order effects. Because we were asking about likelihood to buy, we expected that the ratings would be high for the nonluxury brands overall, regardless of fertility status, due to their relatively lower cost compared to that of luxury brands. However, consistent with hypothesis 1, we expected that the ratings for luxury goods would be significantly higher at high (vs. low) fertility.

To manipulate consumption setting, we stated: “Imagine that you are buying a new T-shirt to wear primarily in front of other people” (public) or “Imagine that you are buying a new T-shirt to wear only at home, and no other people will see it at all” (private). The scores for the six brands in each condition were collapsed and averaged (public-nonluxury: α = .65; public-luxury: α = .97; private-nonluxury: α = .74; private-luxury: α = .99), and this average was the dependent variable. For consistency, we measured SES with the same items as those of Study 1 (*M* = 2.98, *SD* = 1.13; 𝛼 = .71). However, SES did not influence the results. For example, the three-way interaction described below holds when SES is used as a covariate (b = 6.39, *SE* = 2.67, *p* = .017). For this reason, we no longer discuss the impact of SES.

Finally, we assessed each participant’s fertility status. Given the fact that we obtained data from participants across the total duration of their ovulatory cycle, we employed the well-established method of reverse cycle counting in order to determine each participant’s estimated ovulation day [36]. To this end, we asked each participant questions about her ovulatory cycle [8,42]. In particular, participants reported (1) the date their last menstrual period began, (2) the date their second-to-last menstrual period began, (3) the date they expected their next period to begin, and (4) the usual duration of their menstrual cycles. The women also reported how sure they were about each of the four answers (9-point scale; 1 = *Not At All*, 5 = *Somewhat*, and 9 = *Completely*).

We then created the fertility variable by normalizing all participants’ ovulatory cycles onto a 29-day timeframe [36]. We calculated the conception probability (i.e., degree of fertility) for each woman that corresponded to the time during her cycle that she completed the survey [43]. We assigned a value between 0 and.09 (inclusive) to each woman, whereby higher values indicated higher fertility (i.e., higher probability of conception). Previous research [36] supports this as the most reliable method to estimate fertility based on cross-sectional data points.

#### Pretest.

A pretest using 78 women confirmed that the brands were perceived as more or less luxurious, but equally attractive. Participants were instructed to rate how luxurious and attractive (on a 9-point scale) they found six brands. The luxury brands were perceived to be more luxurious (*M* = 7.58, *SD* = 1.29) compared to the nonluxury brands (*M* = 4.63, *SD* = 2.22; *F*(1, 75) = 50.69, *p* < .001, *η*^*2*^ = .40). In addition, attractiveness of luxury (*M* = 6.05, *SD* = 1.57) and nonluxury brands (*M* = 5.66, *SD* = 2.13) did not vary (*F*(1, 76) =.82, *p* = .367, *η*^*2*^ = 01). These results confirm that the brands had the desired characteristics to test our hypotheses.

### Results

**Fig 2:**
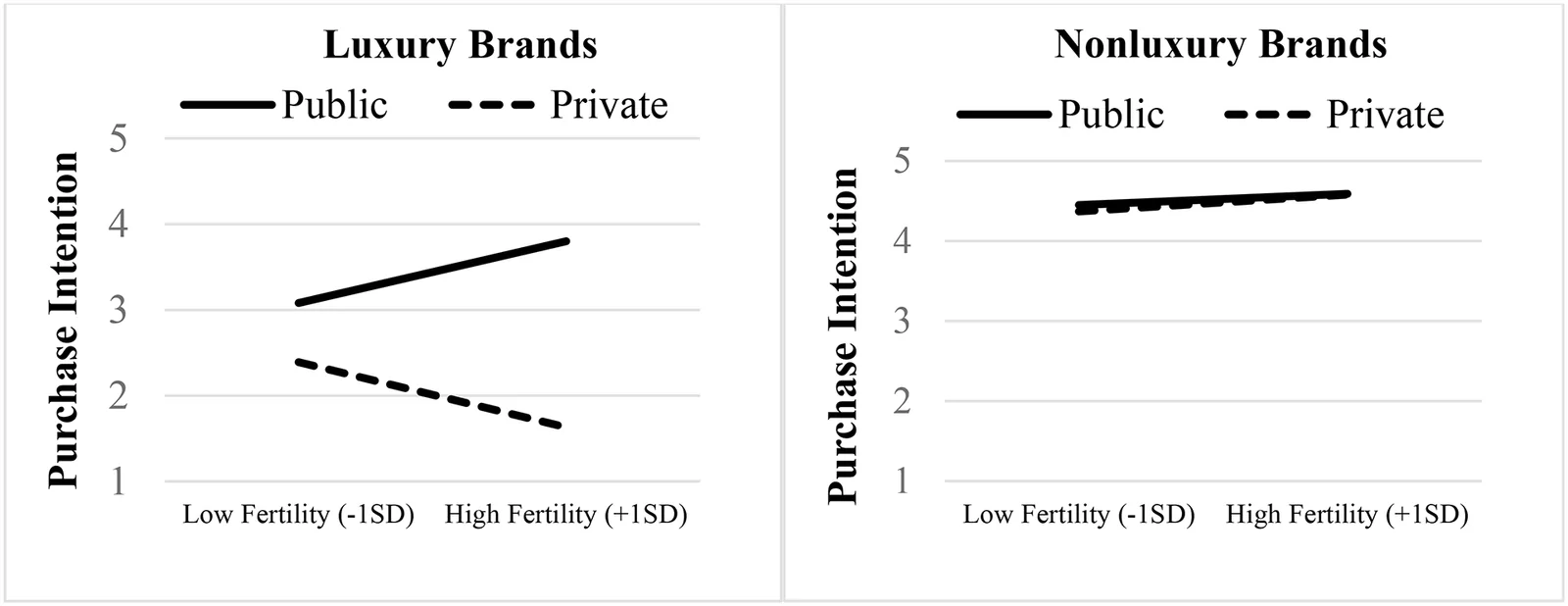
Study 2 Results.

We conducted a regression analysis using fertility (mean-centered), brand type, and consumption setting as the independent variables, and purchase intention as the dependent variable (SPSS PROCESS Macro, Model 3 [44]) (n = 332). We used Model 3 because we expected that the effect of fertility on purchase intentions would be moderated by consumption setting. As predicted, there was a three-way interaction of fertility, brand type, and consumption setting (b = 6.53, *SE* = 2.68, *p* = .015; see Fig 2). An analysis of the conditional effects showed a fertility by consumption setting interaction for luxury brands (b = 12.41, *F* = 9.58, *p* = .002). Fertility had a positive effect on purchase intention in a public setting (b = 12.08, *SE* = 5.47, *p* = .028), such that purchase intention was higher at high fertility (*M* = 3.80) than low fertility (*M* = 3.08). However, fertility had a negative effect in a private setting (b = −12.74, *SE* = 5.87, *p* = .031), such that purchase intention was lower at high fertility (*M* = 1.63) than low fertility (*M* = 2.39). For nonluxury brands, there was no interaction between fertility and consumption setting (b = −.64, *F* = .03, *p* = .856).

The analysis also revealed an interaction between fertility and consumption setting (b = 5.88, *SE* = 2.68, *p* = .029), such that fertility marginally increased purchase intention in a public setting (b = 7.15, *SE* = 3.69, *p* = .054), but not in a private setting (b = −4.61, *SE* = 3.88, *p* = .235). An interaction between brand type and consumption setting was also significant (b = .34, *SE* = .08, *p* *<* .001), such that a public setting increased purchase intention for luxury brands (b = .70, *SE* = .12, *p* *<* .001), but not for nonluxury brands (b = .02, *SE* = .11, *p* = .838). Finally, there was a main effect of brand type (b = −.88, *SE* = .08, *p* < .001), such that participants indicated higher purchase intention for nonluxury than luxury brands, and a main effect of consumption setting (b = .36, *SE* = .08, *p* < .001), such that participants indicated higher purchase intention in a public (vs. private) setting. There were no other effects (*p’s* > .549).

### Discussion

The results of Study 2 support hypotheses 1 and 3, demonstrating that ovulating women have an increased desire to purchase luxury consumed in public, but not in private. This indicates that the increased desire for luxury near ovulation occurs because conspicuous luxury increases the chance that women will be noticed. Purchase intention for nonluxury brands did not change with fertility status, demonstrating that ovulation does not play a role in women’s likelihood to buy brands that cannot be used to signal status.

## Study 3: The moderating role of the belief that men notice women’s designer clothes

Study 3 tests the hypothesis that women do not desire conspicuous luxury near ovulation when they do not believe men notice conspicuous consumption cues (hypothesis 4). If this is the case, then the positive effect of fertility on desire for luxury should be mitigated when women are led to believe that most men do not notice the brand of clothes they wear. Study 3 also limited the sample to women who have recently bought luxury products and employed a real choice as a dependent variable to verify whether the desire for luxury translates to behavior. We asked women to choose between a $100 gift card from a luxury and a nonluxury brand. In addition, all analyses, sample size, and exclusion criteria were pre-registered.

### Method

#### Participants and design.

Prior to starting the study, ethical approval had been obtained from the local institutional review board (IRB) at Rutgers University (# Pro2020000942) to confirm the study met national and international guidelines for research on humans. We collected data from 966 women (*M*_*age*_ = 32.06, *SD* = 5.41) who participated for payment via Amazon’s Mechanical Turk from 2023/12/06–2024/04/06. As in Study 2, this sample size was based on best practice recommendations that cross-sectional, between-subject fertility estimation use a sample size of n = 500 to detect medium size effects [36]. Because the sample size in Study 2 was smaller after exclusions, we now applied several exclusion criteria before the study started (see details below), with the goal of having a larger final sample. All participants viewed written informed consent online prior to starting the survey, and consent was given by completing the survey. The study had a 2-cell (belief: control vs. weak belief that men notice women’s designer clothes) between-subjects design with fertility as a continuous variable.

As preregistered (https://aspredicted.org/C2V_Q4Y), we used the same prescreening criteria outlined in Study 2. However, this time we used a pre-screening tool such that only women who fulfilled the screening criteria participated in the survey. In this study, women who had not bought luxury brands in the past two years were also prescreened. All individuals who successfully completed the prescreening were provided with written informed consent online before they proceeded to fill out the survey. Of the 966 women who fulfilled the pre-screening criteria, 88 women failed the manipulation check and did not indicate having read the correct belief article, and therefore were eliminated from our analysis, resulting in 888 valid responses. We also removed participants who were unsure about their cycle dates (i.e., below a 6 on a 9-point scale) or had abnormal menstrual cycle lengths (i.e., < 25 or > 36 days). The final analysis included 358 women (*M*_*age =*_ 32.73, *SD* = 5.13, ranging 18–40).

#### Procedure.

We first manipulated women’s belief in whether or not men notice women’s designer clothes by asking participants to read a fictitious news article. Participants were told that the first study was about people’s memory for stories and read: “On the next page you will read a recent short article. Please read it carefully one time. You will then be asked a question related to your memory for the story.” Participants in the weak belief condition read an article designed to reduce their belief that men notice women’s designer clothes, entitled “Research Finds What Men Notice the Most.” Those in the control condition read a neutral article entitled “Research Finds There are 2 Trillion Galaxies in the Observable Region of the Cosmos.” Afterwards, we asked participants to indicate, from two options, the main finding of the study they just read. In the control belief condition, the options were: “There are 2 trillion galaxies in the observable region of the cosmos” or “There are 1 trillion galaxies in the observable region of the cosmos”. In the weak belief condition, the options were: “Men notice women’s beauty, smile, and the way they walk” or “Men notice women’s height, table manners, and the brand of the clothes they wear.”

After the belief manipulation, participants were told the next part of survey was on consumer preferences. They read, “We are interested in understanding which brands people prefer when buying clothing items and accessories that they know they will wear primarily in public (i.e., other people will see them and which brand they are). From those who complete this survey, we will randomly select one participant to win a $100 gift card from a certain brand. You can use it to buy any clothing item and/or accessory from the brand. Please choose one option below. If you win, you will receive a $100 gift card from the brand you chose.” Participants chose between a luxury brand (Polo Ralph Lauren) and a nonluxury brand (Old Navy). The order of the brands on the screen was randomized. This choice was the dependent variable. To confirm our belief manipulation worked as intended, participants completed an item: “Do men tend to notice when women wear designer clothes?” on a 7-point scale (1 = *Not At All*, 7 = *Very Much*). Next, we asked questions about menstrual cycles to determine fertility status (fertility was assessed as in Study 2). For consistency with the previous studies, we also measured SES. However, we did not use it in our analyses.

#### Pretest.

A pretest with 67 women confirmed that the brands were perceived as more or less luxurious, but equally attractive. Participants were instructed to rate how luxurious and attractive (on a 9-point scale) they found two brands. Polo Ralph Lauren was perceived to be more luxurious (*M* = 6.09, *SD* = 1.94) than Old Navy (*M* = 2.70, *SD* = 1.69; *F*(1, 66) = 215.17, *p* < .001, *η*^*2*^ = .77). In addition, attractiveness of Polo Ralph Lauren (*M* = 5.58, *SD* = 2.22) and Old Navy (*M* = 5.33, *SD* = 1.98) was similar (*F*(1, 65) =.66, *p* > =.421, *η*^*2*^ = .01). These results confirm that the brands had the desired characteristics to test our hypotheses.

### Results

#### Manipulation check.

We conducted a regression analysis using fertility (mean-centered) and belief (−1 = control, 1 = weak belief) as the independent variables, and perceived belief as the dependent variable (SPSS PROCESS Macro, Model 1) [44]. Participants in the weak belief condition believed that men tend to notice less when women wear designer clothes (*M* = 1.76) compared to those in the control condition (*M* = 3.87, b = −1.06, *SE* = .07, *p* < .001). These results confirm that the belief manipulation worked. There was no other significant effect (*p’s* > .899).

#### Main analysis.

**Fig 3:**
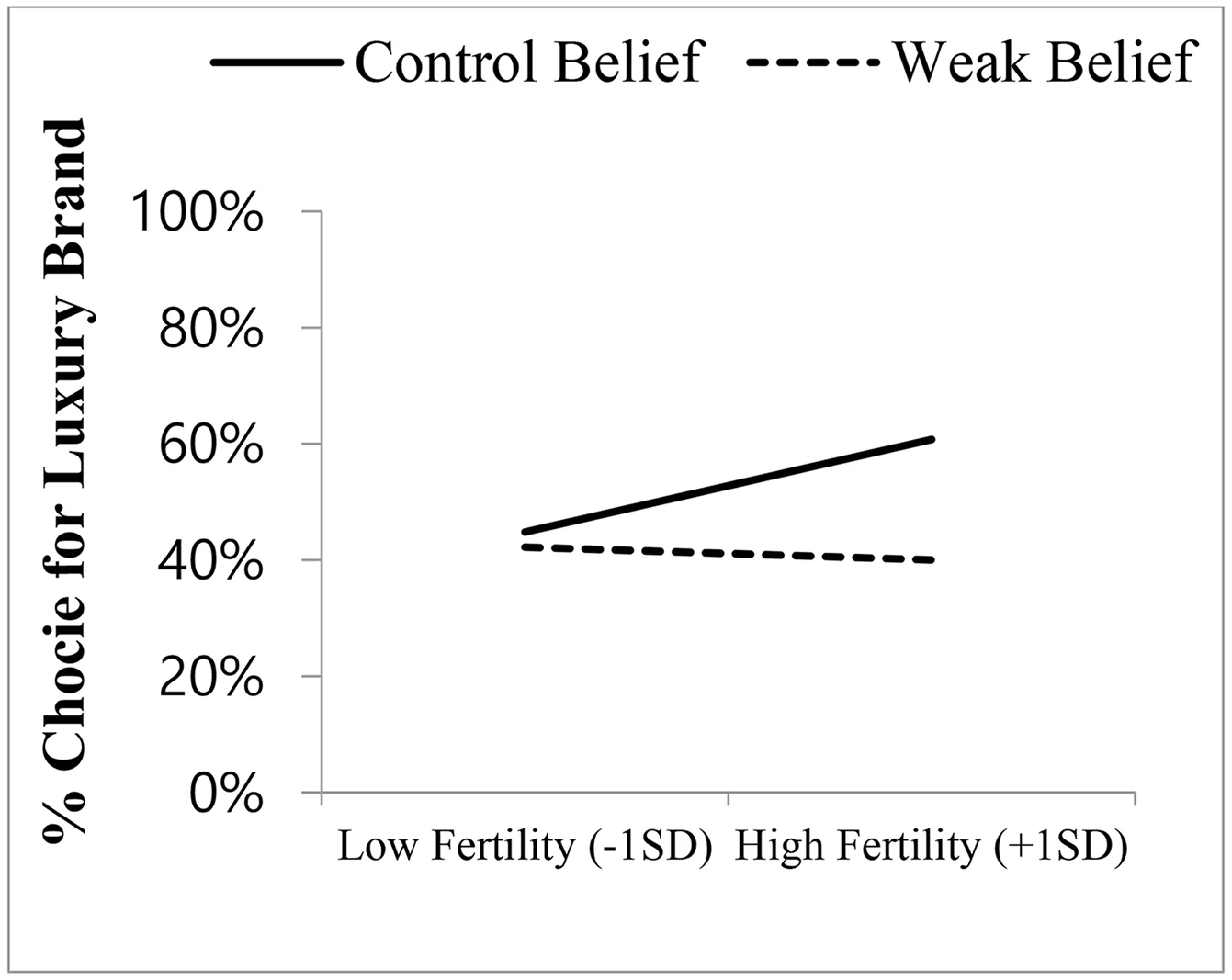
Study 3 Results.

We conducted a similar regression analysis using model 1 and choice as the dependent variable. We used model 1 because we expected that the effect of fertility on choice would be moderated by belief. There was a marginally significant fertility by belief interaction (b = −5.83, *SE* = 3.54, *p* = .099; see Fig 3). Fertility had a marginally positive effect on choice of a luxury (vs. nonluxury) brand in the control condition (b = 10.22, *SE* = 5.56, *p* = .066), such that women chose the luxury brand more often at high fertility (*M* = 60.75%) than at low fertility (*M* = 44.81%). The effect was not significant in the weak belief condition (b = −1.43, *SE* = 4.37, *p* = .744), as women chose the luxury brand similarly often at high (*M* = 40.02%) and low fertility (*M* = 42.20%). There was a negative effect of belief (b = −.24, *SE* = .11, *p* = .029), such that weak belief led women to choose the luxury brand less often than in the control belief condition. There was no other significant effect (*p* = .214).

Looked at differently, at high fertility women chose the luxury brand more often in the control belief condition (*M* = 60.75%) than in the weak belief condition (*M* = 40.02%; b = −.42, *SE* = .16, *p* = .008). The effect was not significant at low fertility (b = −.05, *SE* = .15, *p* = .729), as women chose the luxury brand similarly often in the control (*M* = 44.81%) and weak belief condition (*M* = 42.20%).Given the marginally significant interaction and the high amount of participants excluded due to uncertainty about the menstrual cycle, we also analyzed the results with the full sample, without excluding women who were unsure about their menstrual cycle (n = 888). This analysis showed a significant fertility by belief interaction (b = −4.90, *SE* = 2.16, *p* = .024). Fertility had a positive effect on choice of luxury (vs. nonluxury) brands in the control condition (b = 9.89, *SE* = 3.20, *p* = .002), such that women chose the luxury brand more often at high fertility (*M* = 54.50%) than at low fertility (*M* = 39.02%). However, the effect was not significant in the weak belief condition (b = .09, *SE* = 2.91, *p* = .98), as women chose the luxury brand similarly often at high (*M* = 41.55%) and low fertility (*M* = 41.42%).

### Discussion

The results showed that when women were near ovulation, they were more likely to choose a gift certificate from a luxury brand (Polo Ralph Lauren) over a nonluxury brand (Old Navy). However, when they read about research findings showing that men do not notice women’s designer clothes, they were less likely to choose a luxury brand, even when they were near ovulation. These results support hypothesis 4 and provide further evidence that women who are near ovulation use conspicuous luxury consumption to be noticed by men.

## General discussion

Why do women buy luxury goods? Much of the work on mating-related conspicuous consumption has focused on the factors that motivate men’s desire for luxury goods [12,33]. We proposed that one factor motivating women’s desire for luxury products is the hormonal change that regulates fertility across the cycle. When fertility is high, these hormones can activate a mating goal. Prior work has found that ovulation has important implications for strategic consumption, such as the choice of sexier clothing [8,10,20]. These effects likely occur because ovulation increases women’s desire to optimize the number of potential partners they can choose from [21]. Thus, we reasoned that a desire to expand the pool of mating options near ovulation should lead women to want to stand out and be noticed, which may translate to an increased desire to wear conspicuous luxury products.

We conducted studies using samples of college-aged women and less homogenous nonstudent samples, using both within- and between-subjects methodologies. Consistent with our hypotheses, women who were near ovulation—the time of peak fertility—designed handbags and shoes with larger luxury brand logos for themselves, but not for other women (Study 1). Ovulation was also associated with an increased desire to purchase conspicuous luxury brands, but not nonluxury brands (Study 2). This effect only occurred for luxury products that were to be used in public (Study 2). Finally, the effect did not occur when women were led to believe that men do not notice women’s designer clothes (Study 3). These results have both theoretical and practical implications.

### Theoretical implications

This work makes an important contribution to the literature on conspicuous consumption [2–3] and how biological factors affect consumer preferences [8,9,12,45–48]. The idea that fertility should influence women’s desire for luxury products is not straightforward because luxury goods do not typically enhance a woman’s physical attractiveness. However, luxury products provide a means to stand out compared to other women. This is important because, athough broader work shows that conspicuous luxury signals status and prestige to others generally [4,28], prior research on mating motives and luxury consumption has focused primarily on men [12,33]. Our findings address this gap by revealing how mating motives shape women’s luxury purchasing.

The existence of an attraction phase and a selection phase is an important issue that has not received much attention in the literature. This distinction is important as it directs attention to the need to better understand context with respect to mating-motivated behavior. For example, some findings indicate that a mating motive leads women to exhibit traits related to kindness [33], which occurred in contexts where the women had found a desirable mate. Other findings indicate that women may want to conform to their partner’s preferences [49], which occurred in the context of serious relationships. Thus, while many findings show different types of effective behaviors that women may use to satisfy what men desire from a woman, it is important to consider the effectiveness of strategies aimed at being noticed by men, before finding a desirable partner.

We have proposed that these strategies will be overall effective if women are not noticed for negative reasons. For example, dressing in a sexier way when other women are around [10] is an effective strategy to outshine other women, working both to be noticed and to make men feel attracted. This begs the question of what it is about conspicuous luxury signals that could attract men, beyond being noticed.

We speculate that a mating-motivated ovulatory shift in luxury consumption can be effective beyond being noticed, both from women’s and men’s standpoints, for several reasons. First, this behavior is not due to an attempt to show dominance or the possession of financial means, as these behaviors may be unattractive to some men [32,50,51]. However, these behaviors may be due to wanting to show status, which has positive associations. Being seen as someone who has status and prestige sends a signal about a woman’s ability to be successful in life. This success may involve different aspects of life, such as her ability to solve problems, manage finances, or educate kids. Thus, these signals should increase a woman’s ability to outshine other women and attract desired mates. Second, beyond being noticed, luxury products usually look better than nonluxury products, following the latest trends. This aesthetic advantage should make a woman more attractive, just like other items not related to luxury (e.g., hair, skin). Third, we live in a society that values possessions such as designer clothes and luxury products in general. Women, like men, certainly value these possessions. Thus, a woman wearing luxury items may feel an advantage over other women who do not wear such items. Having an advantage on a factor that is visible to others should make women themselves feel more valuable, resulting in confidence that would be attractive to men.

Also, the type of luxury we investigated were popular categories, like t-shirts, bags, and shoes. Beyond the aesthetic advantage of luxury products, popular items that are desired by people (i.e., luxury) and are high in quality (better materials, better stitching) are more likely to feel like they are the best or one of the best options in a category. Related to the confidence discussed above, wearing one of the best options should make women feel more secure, which should also be attractive to men. This is especially useful given that the level of luxury in our investigation is within easier reach than other types of luxury, like expensive furniture or a high-end vehicle. Thus, the strategy can be used with some frequency. In summary, this level of conspicuous luxury is likely to be noticed by men, is relatively accessible, and there are many possible ways in which wearing it sends a positive signal that attracts a man.

While we have investigated luxury consumption, it is possible that women try to outshine other women in different domains. For example, they may try to be better than others at sports, have a higher-quality and more intense social life, or even have better health, which has an evolutionary advantage [52]. Whether women are likely to show these behaviors depends on their beliefs about these behaviors – would they be noticed by men? This may depend on how visible the signal is. While better skin health, for example, should be noticed, a well-functioning kidney should not play a role in attracting a mate. While a high-quality social life should make most of us happy, this strategy would only be used and effective at attracting men if men were present at these events and noticed the woman. A similar argument can be made about doing well at sports. Future research should examine alternative behaviors women may use in the initial attraction phase, with a focus on how visible and desirable these behaviors are. If a behavior is visible and is not seen negatively, it should be effective as an initial way to be noticed by men, to then allow for a selection process to take place.

### Practical implications

These findings have implications for both luxury and nonluxury brands. Luxury brands can emphasize the social benefits of being noticed in their messaging. Conversely, nonluxury brands may benefit from positioning themselves as offering status through subtlety—highlighting the appeal of avoiding conspicuous logos and “trying too hard.” This represents a departure from existing research, which has largely overlooked strategic implications for nonluxury brands. Beyond competing on traditional attributes like price, nonluxury clothing brands could emphasize the social advantages of understated style.

Nonluxury brands could test two parallel social media campaigns—one emphasizing affordability, another emphasizing effortless style and logo-free design. Standard engagement metrics (likes, shares, follows) could be tracked, with additional analysis by gender and timing.

The current research suggests that campaign performance may vary by gender and ovulatory cycle phase. During peak fertility, women may respond more strongly to visibility-focused messaging (favoring the price campaign over the “effortless style” campaign), while during other cycle phases they may prefer subtlety-focused messaging (favoring the “effortless style” campaign). Tracking these patterns could allow brands to optimize ad targeting based on cyclical variation in consumer preferences.

Alternatively, this research has implications for communication strategies in luxury markets, which do not typically advertise “being noticed” as one of their advantages. In fact, our findings are novel because they emphasize the advantages of being noticed in certain contexts, whereas previous research has emphasized that many consumers do not want others to notice their luxury consumption [31]. Still, if being noticed were not important to a sizeable number of people, there would be no need for logos and other symbols associated with luxury brands (e.g., the Louis Vuitton logo, the Louboutin red soles). The implication is that highlighting the idea of being noticed may be too obvious and backfire, but there are subtle ways of implying that men will notice women wearing conspicuous luxury.

For example, an ad could highlight two women at a party wearing similar outfits, but one clearly shows the brand, and this would be the woman men’s heads turn toward. Or, a social media post could show men being exposed to different colors and patterns, but stopping when they see something a brand is known for (e.g., the Gucci red and green stripes). In addition to actual buying behavior, one way to test the effectiveness of such strategies is to ask consumers not just how much they noticed certain logos/brands, but also aspects of the women wearing them. Examining the transfer of attention from the brand to the consumer, and communicating this transfer in future campaigns, can increase the resonance of these marketing appeals among women.

At the very least, luxury brands should be more aware that the women being exposed to their marketing efforts face hormonal changes and be strategic about how to position their products based on how these changes influence desires and behaviors. Understanding changes that occur to a large proportion of the female population, for a significant part of the month, offers opportunities for brands. As an additional example, the concept of being noticed is typically associated with being seen by other people. Brands may want to expand this idea by communicating to female consumers about *standing out*, beyond just being seen. This could mean hiring a female spokesperson who stands out for speaking eloquently, which would signal to women that the brand is for women who stand out. Another possibility is to have an ad campaign “for women who stand out for more than looks”. As discussed earlier, women may be noticed for many reasons. Thus, luxury brands may want to explore opportunities beyond the idea of wearing those brands to be seen, helping women stand out for other qualities.

### Future research

In addition to the research opportunities discussed above, future work may wish to examine factors that moderate the focal effect, with regard to women’s behavior as buyers and in general (Otterbring et al., 2020). For example, women’s desire for luxury may also change depending on how competitive the local mating market is [53–54]. When there are more females than males, women’s interest in luxury products may be even higher. This would occur because the presence of many women would make the motive to outshine them even more salient. While anecdotal evidence suggests that women are more likely to wear conspicuous luxury to social events, it would be premature to affirm that this is driven by wanting to be noticed by men. Yet, this motive can play an important role, and future research may want to investigate which proportion of luxury consumption is driven by each of several possible motives.

Another possible moderator is the phase in the mating process where the women are situated. In our studies, women where ovulating or not, which can activate a mating goal. But unlike other research, they were not put in scenarios where they had found a desirable partner, were not near specific women that they wanted to compete with, and were not making a specific selection in a context where there were already (more than) enough alternatives [11]. Our effect is more generalizable in the sense that if women are near ovulation, the motive to be noticed by men should be active, which should increase desire for conspicuous luxury. Thus, the women in our studies were not trying to attract men during the study per se, but they were experiencing hormonal changes that influenced their behavior. Yet, it is important for future research to examine the role luxury plays in different phases of the attraction and relationship development process, from the perspective of both men and women [32]. Although some work has found that behavior can be influenced through scenarios (e.g., “imagine you are at a party and found someone you like”) [21,55], stronger effects may be evident for contexts that are somewhat real, for example by using confederates that most women would be attracted to [42,56].

Another question is what happens when women are trying to attract other women. While our conceptualization and studies focus on women’s behavior when trying to attract men, we recognize the existence of different contexts where these results may or may not apply, such as when women may want to attract other women. Homosexual women ovulate and experience the same hormonal fluctuations across the cycle. Thus, the motivations and behaviors we investigated would likely be present in homosexual women, although the target would be other women. Supporting these ideas, lesbian women who were near ovulation dressed in a sexier way [8,57], which suggests that they would show a similar desire for luxury as heterosexual women near ovulation. In addition, we measured sexual orientation in Studies 1 and 2, and found that on average 86% (14%) of our sample was (not) heterosexual. In both studies, controlling for this variable did not change our results, indicating that the effect holds across different sexual orientations.

The signals examined in the present research may serve a dual audience. The ultimate goal of being noticed is to find a suitable parter, a goal that can also be served by having fewer women to compete with or showing superiority to other women. Consistent with this view, prior research demonstrates that ovulating women desire superior products relative to other women, behave less cooperatively with female partners in economic games, and dress more provocatively in the presence of other women [8,17,58]. Beyond ovulatory effects, women’s conspicuous luxury consumption can also function as a rival-deterrence signal directed specifically at other women [42]. Importantly, intrasexual competition is an individual difference variable, with some women being more likely than others to see other women as their competitors [59]. This presents an interesting perspective for future research. If women want to be noticed by men independently of the presence of other women, then intrasexual competition as an individual difference should not matter. Alternatively, if women wear conspicuous luxury partly because they see other women as competitor, than the effect should be stronger for women who are high on intrasexual competition, but attenuate for those who are low on intrasexual competition.

These are possible moderators associated with mating, but motivations other than mating may shift women’s luxury consumption [60]. For example, parenting motivations may shift attention from self-focused consumption to child- or family-focused consumption that signals ability to succeed as a parent or cooperative partner. This is a fascinating research question as we know parents like to stand out in terms of their parenting skills. No research, however, has looked at whether conspicuous luxury consumption may be used to signal such skills. We hope our conceptualization and findings will spur future work on fundamental motives, hormones, and women’s consumer behavior.

### Limitations

This research has limitations. First, we used a convenience sample of women who signed up to participate in our studies. While there are advantages to doing so when using an experimental research paradigm (i.e., it allows for better control of the influence of external variables), it would be beneficial to examine whether the findings apply to a representative sample of the female population. Relatedly, there was a high level of exclusion due to the fact that many participants did not meet the criteria to participate or were unsure about their menstrual cycle. While this follows accepted procedures in this field of study, this is a limitation in the sense that a lot of resources are used (e.g., payment for participation) on participants whose data are not useful.

Second, we conducted this investigation in the contexts of logo sizes and preference for luxury brands. Three studies showed insightful and consistent results, but we cannot claim that these findings apply to every other context where women’s goal is to attract men. In fact, the contexts we used were general, where women were ovulating (or not) and were asked to indicate their preferences. The findings may not apply, for example, in a context where women are at a public place with friends, and do not want to compete with their own friends for men’s attention. Or, there are contexts and communities in which luxury is simply not appreciated, in general, and we do not claim that our findings would apply in these contexts.

Third, there is a claim to be made that not all of our findings are causal. In the contexts of experiments, where participants are randomly assigned to conditions, it is possible to infer causality. However, study 1 is entirely within-subjects, and therefore there were no conditions to randomly assign participants to. In addition, hormonal status (a high versus a low fertility state) is akin to a state-level individual difference variable, like hunger. Studies looking at the effects of ovulation on behavior are not causal in the strict sense applied in experimental research. To help mitigate this issue, a within-subjects design using hormone tests, as used in Study 1, is considered the best way to strengthen claims of causality in hormone research. This is the case because each person in a within-subects design serves as their own control, ruling out situational or individual differences as accounts for the effects [36].

Despite these limitations, the overall evidence provides strong support to the idea that women’s ovulatory cycle can influence their behavior toward men, especially with regards to using conspicuous luxury symbols in order to be noticed. We hope future research can expand on these findings, adding insights to what we already know about how women and men behave when trying to find partners for a romantic relationship.

## Supporting information

S1 File Appendix A. Demographic Characteristics for Studies 1 and 2. Appendix B. Stimuli for Study 3. (DOCX)
