# Supplementary material for: Fertility increases women’s desire for conspicuous luxury
Source: PLoS One. 2026 Sep 2;21(9):e0354126. doi: 10.1371/journal.pone.0354126 (PMC13537554; doi:10.1371/journal.pone.0354126)
Supplement: Supplementary file 1 [file pone.0354126.s001.docx]

**Appendix A. Demographic Characteristics for Studies 1 and 2**

**Study 1 Demographics**

| Age | Mean | SD |
| --- | --- | --- |
|  | 22.59 years | 5.09 years |

| Income | $15,000 or less | 20.3 % |
| --- | --- | --- |
|  | $15,001 - $25,000 | 18.8 % |
|  | $25,001 - $35,000 | 11.6 % |
|  | $35,001 - $50,000 | 11.6 % |
|  | $50,001 - $75,000 | 11.6 % |
|  | $75,001 - $100,000 | 13.0 % |
|  | $100,001 - $150,000 | 5.8 % |
|  | $150,000+ | 7.2 % |

| Ethnicity | Asian/Asian-American, Chinese, Japanese, and others | 11.4 % |
| --- | --- | --- |
|  | Black/African-American | 12.9 % |
|  | Hispanic/Latino, Mexican-American, Central-American, etc. | 40.0 % |
|  | White/Caucasian/Anglo/European American | 28.6 % |
|  | American Indian/Native-American | 2.9 % |
|  | Mixed (my parents are from different ethnic groups) | 4.3 % |

| Relationship Status | Married | 7.1 % |
| --- | --- | --- |
|  | Living with my partner | 14.3 % |
|  | Engaged | 4.3 % |
|  | Dating/involved with only one partner | 30.0 % |
|  | Dating/involved with more than one partner | 2.9 % |
|  | Not dating/romantically involved with anyone | 38.6 % |
|  | Other | 2.9 % |

**Study 2 Demographics**

| Age | Mean | SD |
| --- | --- | --- |
|  | 30.79 years | 6.39 years |

| Ethnicity | Asian/Asian-American, Chinese, Japanese, and others | 10.8 % |
| --- | --- | --- |
|  | Black/African-American | 13.0 % |
|  | Hispanic/Latino, exican-American, Central-American, etc. | 6.3 % |
|  | White/Caucasian/Anglo/European American | 63.9 % |
|  | American Indian/Native-American | 0.3 % |
|  | Native Hawaiian/Pacific Islander | 0.3 % |
|  | Mixed (my parents are from different ethnic groups) | 4.8 % |
|  | Other | 0.6 % |

| Relationship Status | Married | 39.8 % |
| --- | --- | --- |
|  | Living with my partner | 7.2 % |
|  | Engaged | 4.5 % |
|  | Dating/involved with only one partner | 18.7 % |
|  | Dating/involved with more than one partner | 0.6 % |
|  | Not dating/romantically involved with anyone | 28.6 % |
|  | Other | 0.6 % |

**Appendix B. Stimuli for Study 3**

**Research finds there are 2 trillion galaxies in the observable region of the cosmos.**

By MORGAN JAMESTON, Senior Times Writer

The earliest estimates of the number of galaxies in the Universe were very small. Most recent estimates built off observations from 1995, when NASA’s Hubble Space Telescope stared at a dark patch of sky for hours and returned a picture of thousands of glittering galaxies no one had ever seen before.

**Research finds what men notice the most.**

By MORGAN JAMESTON, Senior Times Writer

Recently, a survey with 3,000 men in the US examined what men notice and do not notice in a woman. The great majority of men indicated that they notice women's beauty, smile, and the way they walk. In contrast, the great majority of men indicated that they do NOT notice women's height, table manners, and the brand of the clothes they wear.
